# Profiling genome-wide recombination in Epstein Barr virus reveals type-specific patterns and associations with endemic-Burkitt lymphoma

**DOI:** 10.1186/s12985-022-01942-8

**Published:** 2022-12-08

**Authors:** Eddy O. Agwati, Cliff I. Oduor, Cyrus Ayieko, John Michael Ong’echa, Ann M. Moormann, Jeffrey A. Bailey

**Affiliations:** 1 Department of Zoology, Maseno University, Maseno, Kenya; 2 Center for Global Health Research (CGHR), Kenya Medical Research Institute, Kisumu, Kenya; 3 Department of Pathology and Laboratory Medicine, Warren Alpert Medical School, Brown University, Providence, RI 02903 USA; 4 Program in Molecular Medicine and the Diabetes Center of Excellence, University of Massachusetts Chan Medical School, Worcester, MA 01605 USA

**Keywords:** Epstein–Barr virus, Genome-wide recombination, Endemic-Burkitt lymphoma

## Abstract

**Background:**

Endemic Burkitt lymphoma (eBL) is potentiated through the interplay of Epstein Barr virus (EBV) and holoendemic *Plasmodium falciparum* malaria. To better understand EBV’s biology and role in eBL, we characterized genome-wide recombination sites and patterns as a source of genetic diversity in EBV genomes in our well-defined population of eBL cases and controls from Western Kenya.

**Methods:**

EBV genomes representing 54 eBL cases and 32 healthy children from the same geographic region in Western Kenya that we previously sequenced were analyzed. Whole-genome multiple sequence alignment, recombination analyses, and phylogenetic inference were made using multiple alignment with fast Fourier transform, recombination detection program 4, and molecular evolutionary genetics analysis.

**Results:**

We identified 28 different recombination events and 71 (82.6%) of the 86 EBV genomes analyzed contained evidence of one or more recombinant segments. Associated recombination breakpoints were found to occur in a total of 42 different genes, with only 7 (16.67%) being latent genes. Recombination events were major drivers of clustering within genome-wide phylogenetic trees. The occurrence of recombination segments was comparable between genomes from male and female participants and across age groups. More recombinant segments were found in EBV type 1 genomes (*p* = 6.4e − 06) and the genomes from the eBLs (*p* = 0.037). Two recombination events were enriched in the eBLs; event 47 (*OR* = 4.07, *p* = 0.038) and event 50 (*OR* = 14.24,* p* = 0.012*)*.

**Conclusions:**

EBV genomes have extensive evidence of recombination likely acquired progressively and cumulatively over time. Recombination patterns display a heterogeneous occurrence rate across the genome with enrichment in lytic genes. Overall, recombination appears to be a major evolutionary force impacting EBV diversity and genome structure with evidence of the association of specific recombinants with eBL.

**Supplementary Information:**

The online version contains supplementary material available at 10.1186/s12985-022-01942-8.

## Background

Epstein Barr virus (EBV) is a ubiquitous gamma-herpesvirus from the family of primate lymphocryptovirus (LCVs) [1]. EBV primarily infects B lymphocytes and epithelial cells [2] and over 90% of the global human population have contracted EBV by adulthood and carry the latent virus lifelong [3, 4]. While EBV infection is asymptomatic long-term in the vast majority, it still accounts for significant morbidity and mortality, with over 1% of global cancers being associated with the virus [5]. In sub-Saharan Africa (SSA), EBV and repeated long-standing *Plasmodium falciparum* (*Pf*) malaria infections are associated with a markedly increased incidence of endemic Burkitt lymphoma (eBL) [6], which is an aggressive non-Hodgkin B cell lymphoma that affects the pediatric population [7]. *Pf* malaria may contribute to increased eBL in multiple ways including promoting polyclonal B cell expansion [8]; affecting viral reactivation and host immune clearance of EBV-infected cells; [6, 9], and increasing activation-induced deaminase (AID) DNA damage, all of which would likely increase the chances of the c-Myc translocation, the hallmark of eBL development [10]. Studies have identified EBV in almost all eBL tumors (≥ 90%) from malaria-endemic regions in Africa [5] suggesting an integral role in tumorigenesis, however, the exact mechanism(s) of EBV involvement is not fully understood. EBV-associated malignancies are characterized by strong geographic differences in prevalence [11] with eBL prevalent in African populations in SSA [12] while nasopharyngeal carcinoma (NPC) is highly prevalent among the Southeast Asian population [13]. These geographic differences may be attributed in part to cofactors including the host genetic factors, viral variations, and environmental factors [11]. Studies probing host genetic factors implicated in EBV-associated malignancies have identified several human leukocyte antigen (HLA) alleles associated with susceptibility to NPC among the Southern Chinese population [14, 15]. Since EBV and *Pf* infections are known contributors to eBL-associated malignancies, eBL is influenced indirectly by environmental factors such as climate, rainfall, and vegetation that affect the burden and transmission of *Pf* [16]. Further, the age of primary infection and severity of EBV infection is influenced by the lifestyle of the inhabitants with the majority of children being infected by EBV within their first years of life [17].

The EBV genome measures approximately 172 kb and has at least 86 open reading frames (ORFs) [4]. Nine ORFs encode the key latent proteins including EBNA-1, EBNA-2, EBNA 3A, -3B, -3C, EBNA-LP, LMP-1, LMP2A, and -2B [18, 19]. Key latent genes (EBNA 2 and EBNA3s) harbor deep-seated amino acid variation that defines type 1 and type 2 [4]. Other ORFs encode capsid proteins, transcriptional factors, lytic proteins as well as non-coding RNAs [4]. EBV-associated gene products play various roles in EBV infection, cell-to-cell spread, and the transformation of host cells [18, 19] among other roles critical in EBV’s biology, therefore acquired variations within these genes could alter or enhance EBV pathogenic potential in infected cells leading to the development of EBV-associated diseases. Deep-seated variation underlying EBV type 1 and 2 genomes as well as abundant variations acquired elsewhere in the genome has motivated the EBV research community to sequence the viral genome and determine if viral variation impacts disease risk.

Advances in targeted enrichment and DNA sequencing technology have greatly improved our knowledge of EBV genomic variation [20–23]. It is clear now that in addition to point mutations, recombination is a key force in shaping viral variation [24]. Studies have identified SNPs resulting from point mutations that may increase the risk of EBV-associated malignancies such as NPC and eBL [5, 11, 13, 25–27]. Further, EBV has been genotyped as a Type 1 and Type 2 virus based on deep-seated divergence in variations in the *EBNA 2* gene and EBNA 3 family of genes [4, 28]. Type 1 EBV has been shown to be better at immortalizing B cells in vitro [29, 30] and was recently implicated in the development of eBL [21]. However, to the best of our knowledge, no studies have identified recombination signatures that may increase the risk of EBV-associated conditions.

Genetic recombination occurs when two genomes co-infect the same cell and exchange genetic fragments leading to genomic rearrangements [31]. When this process occurs, it can create a variant profile that may increase the risk of disease [31]. For example, recombination events in *EBNA 3* genes lead to changes that affect their immunogenic determinants providing a route for EBV immune escape [32]. Berenstein et al. [33] reported a highly variable landscape of recombination rates along the EBV genome patterns which may underlie key biologic features of EBV. Studies in other herpesviruses such as the herpes simplex virus (HSV1) support the important role of recombination, with breakpoints in genes that were associated with better capabilities to evade host immune surveillance [34]. The role of recombination in altering the risk of EBV-associated malignancies is unclear given the lack of a properly controlled investigation involving cases and controls.

To address the role of recombination in eBL, our study examined publicly available EBV sequences which we previously obtained from the viral DNA of eBL patients and geographically matched healthy controls from a geographic region in western Kenya [21]. This region with a high incidence of eBL [35] is characterized by holoendemic *Pf* malaria [36], early age of EBV infection [37], and extensive co-circulation of EBV type 1 and type 2 [21]. Using a computational approach, we investigated if recombination patterns created variant profiles that could influence the pathogenic potential of EBV type 1 and type 2 genomes leading to their relative tumorigenicity. Further, we characterized the landscape of recombination in EBV genomes from the healthy and eBLs to identify recombination signatures that may augment eBL pathogenesis.

## Methods

### EBV sequence datasets

We used 108 EBV sequences available in the European Nucleotide Archive (http://www.ebi.ac.uk/ena), under the study accession no. ERP122181, which were downloaded in FASTA format. Within this set, 4 long-term laboratory strains (Jijoye, Raji, Namalwa & Daudi), 6 patient plasma samples, and 3 patient-derived cell lines, as well as 1 eBL case and 8 healthy controls with poor coverage (< 50%), were excluded as our aim was to include high-quality virus sequences directly obtained from patient tumors and healthy controls. The final dataset of 86 genomes (Additional file 4: Table S1) was comprised of 54 confirmed eBL cases diagnosed at Jaramogi Oginga Odinga Teaching and Referral Hospital (JOOTRH), the referral center for children diagnosed with cancer in western Kenya [36], and 32 geographically matched healthy children with no history of cancer that resided in the same geographical area (Kisumu County) as the eBL cases. The corresponding participant data included the age, viral type, and gender.

### Multiple sequence alignment

The 86 samples (54 eBL cases and 32 geographically matched controls) were aligned using MAFFT software version 6 [38] engaging the automatic algorithm with default parameters. All the resulting multiple sequence alignments (MSAs) were manually inspected using PhyloSuite v1.2.2 [39]. Since poorly aligned regions, with excessive alignment gaps, can generate artificial genomic diversity, we used Gblocks to trim the alignments ensuring the downstream phylogenetic inferencing was performed on genomes with reliable alignments and thus avoiding any artificial genomic diversity [40]. We preferred GBlocks because it uniformly trims aligned sequences at the same positions and allows researchers to reproduce the same final alignments. After the gblocks exclusion, 51% (88 kbp) remained (Additional file 7: Trimmed MSA) on par with previous multiple alignment analyses that examined 48% of the genome [32]**.**

### Phylogenetic inference

The trimmed MSA was then subjected to phylogenetic analyses using molecular evolutionary genetics analyses version 7 (MEGA 7) [41]. The phylogenetic tree was constructed using the neighbor-joining (NJ) algorithm, and evolutionary distances were computed using the Jukes-Cantor model with ambiguous nucleotides removed by pairwise deletion. Bootstrap analyses of 5000 replicates were performed on each tree to determine confidence and the final tree was rooted in the midpoint branch.

### Detection of recombination

Rapid recombination program (RDP4) [42] was used on the trimmed MSA to detect recombinants and breakpoints with an ensemble of methods including both phylogenetic methods (Bootscan and RDP) and substitution methods (Chimaera, GENECONV, MaxChi, Siscan, and 3Seq). Maintaining the default window and step sizes at 200 and 20 respectively, the RDP4 methods scanned the aligned genomes and provided a detailed output of recombination events detected coded with unique numbers, sequences with evidence of such events, and the coordinates of the corresponding breakpoints in the MSA (Additional file 5: Table S2). Putative recombinant events were only considered when all the six algorithms (RDP, GENECONV, Chimera, Maxchi, 3Seq, Bootscan, and Siscan) identified the recombination event and had a threshold *p* value of 0.05, using Bonferroni correction.

To assess the reproducibility of event calls, we characterized and compared recombination patterns in genomes obtained from 6 plasma specimens along with their tumor biopsy replicates. Since the viral DNA in the plasma has been shown to be a representative of the virus in the tumor cells [21], they should therefore share similar recombination patterns. We demonstrate the same recombination events in the plasma-tumor replicates (Additional file 1: Fig. S1). This approach allowed us to confirm the precision of our in silico method to characterize recombination signatures within the population.

### Genomic feature annotation

The coordinates of the recombination events and their breakpoints were mapped to the EBV type 1 reference genome (GenBank accession NC_007065). Annotated genomic features including gene positions, coding regions, introns, as well as regulatory regions corresponding to the recombination signatures were extracted from the reference genome BED format file and visualized using integrative genome viewer (IGV) [43].

### Statistical analysis

Further statistical analyses were performed using R statistical software (Version 3.6.1) [44]. Wilcoxon rank test was used to compare the signatures of recombination between viral types and between eBL and healthy cohorts. Fisher exact test was used to test EBV type association with unique recombination events and their breakpoints. Univariate and multivariate logistic regression modeled eBL association with recombination events and their breakpoints. Statistical significance was defined at *p* < 0.05.

## Results

### Demographic characteristic of study participants

We examined 54 confirmed eBL cases and 32 healthy controls with well-assembled viral genomes which were previously sequenced and examined for single nucleotide variation. The general characteristics of the study participants are summarized in Additional file 6: Table S3, and were consistent with known features of eBL including increased incidence in males 74% (40/54) and type 1 EBV being more prevalent (70.9%) [21, 45]. The participants were stratified into age groups i.e. 0–4, 5–9 and 10–14 years, as previously done [46]. This stratification was based on the temporal relationship between EBV infection, *Pf* malaria infection, and the occurrence of eBL in children from western Kenya [47]. More BL-positive children were aged 5–9 years (57.4%) consistent with the peak incidence of eBL occurrence [36]. More healthy controls were aged 0–4 years (90.6%) and none above 10 years, as the younger children have high EBV loads [46] required for sequencing [48, 49].

### Evidence of recombination in EBV

Recombination events were detected across all the 86 high-quality genomes using RDP4 following multiple alignment. After filtering well-supported recombination events detected by all six RDP4 methods, we retained 28 distinct recombination events (Additional file 2: Fig. S2). Of the 86 genomes, 82.6% (71/86) contained at least one breakpoint and the average number of recombinant breaks in each genome was 3.5 (median = 4, *range* = 0–8) with no genome representing heavy mosaicism compared to the others. This level of recombination between genomes from western Kenya is on par with previous reports in EBV from other geographical regions [23, 32] and consistent with other herpesviruses such as herpes simplex virus (HSV) [50], murine cytomegalovirus (MCMV) [51], and human cytomegalovirus (HCMV) [52].

### EBV diversity and population structure related to recombination patterns

Recombination events with the potential to exchange large regions of the genome can dramatically modify a genome affecting phylogenetic relationships and importantly biology [53, 54]. We first examined recombination events based on how often they were observed within our sample population. Interestingly, the minority of events, 32.1% (9/28) were detected in only one genome, while the majority 67.9% (19/28) were present in two or more genomes. Many were common with a quarter of all events present in 8 or more genomes and likely represent evolutionary distant recombinant events that have propagated extensively over time.

**Fig. 1:**
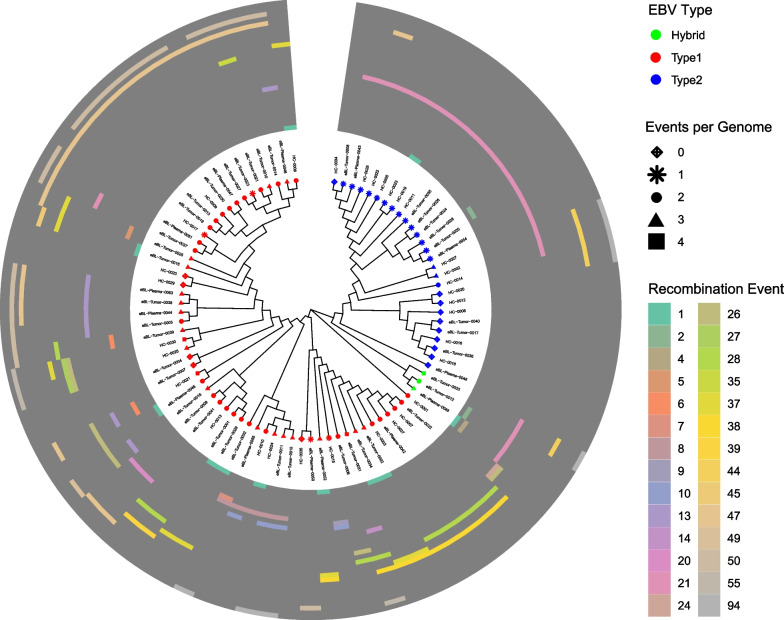
Phylogenetic tree of EBV isolates showing diversity related to genomic recombination events. The analysis involved 86 EBV genome sequences. The evolutionary history was inferred using the NJ method. Evolutionary distances were computed using the Jukes–Cantor model. Ambiguous nucleotides were removed using pairwise deletion. Bootstrap analysis of 5000 replicates was performed. A circular heatmap was used to annotate the tree using the 28 recombination events detected

We thereafter examined recombination events in relation to phylogenetic relationships constructing phylograms from the nucleotide variation within multiple alignments and annotating recombination events on the phylogram branches based on genomes sharing the same events within a clade (Fig. 1). The first major division in the tree was between type 1 and type 2 viruses consistent with previous observations of the significant dichotomy between types [20, 21, 24]. While 33.3% of type 2 genomes (10/30) had no evidence of recombinant segments, 91.1% (51/56) of type 1 genomes (Fig. 1) had one or more segments. Despite most recombination events, 67.9% (19/28) appearing in multiple isolates, the recombinant segments were seldom shared between type 1 and type 2 genomes; For instance, event 21 was only observed within type 2 genomes, and events; 28, 37, 38, 47, and 50 were exclusively in the type 1 genomes. In general, the events shared between multiple isolates, clustered by phylogenetic clades, suggest that recombinant segments shared by multiple isolates drive a significant portion of the phylogeny and appear propagated from a common ancestral recombination event. The clustering of isolates in the type 2 branch was distinct to give two recombinant phylogroups. The first phylogroup consisting of 9 isolates had no evidence of recombination signatures and was much closer to the typing branch. The second phylogroup consisted of 16 isolates with evidence of recombination event 21 convened distinctly away from the isolates of the first phylogroup. This correlated with a previous observation where type 2 genomes demonstrated novel substructures [21]. Together, these suggest that recombinant events are a significant driver of substructure both within and between the known viral types.

### Recombination patterns between EBV types

**Fig. 2:**
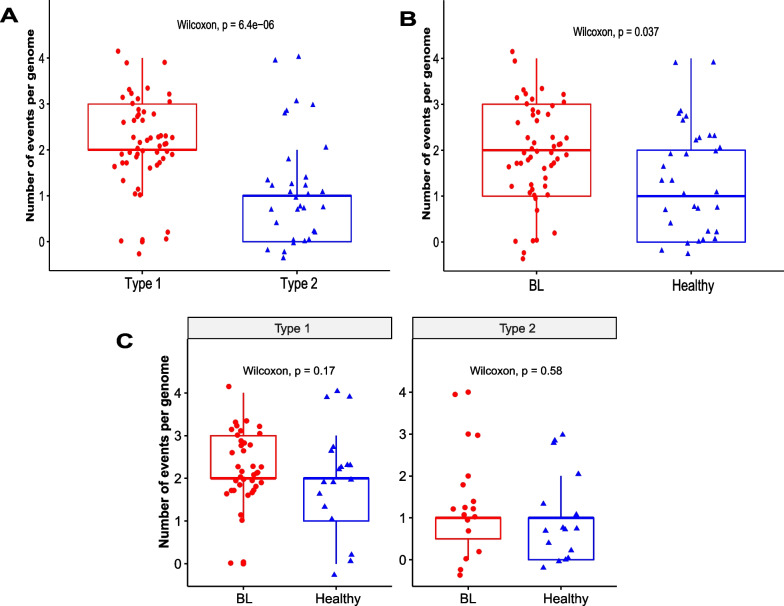
Recombination events per genome stratified by **A** Viral Type, **B** BL Status, and **C** Viral Type Relative to BL Status. Center Lines represent medians, with lower and upper boundaries of the boxes representing first and third quartiles respectively. Wilcoxon (**A**, **B** and **C**) tests were performed and *p value* < 0.05 was considered significant

As EBV types are the major molecular classification within EBV [4] we sought to further compare and contrast the patterns of recombination in type 1 and 2 viruses to better understand the role of recombination (Table 1). Statistical tests showed specific recombinant segments that were enriched among type 1 genomes: events 28, 37, 38, 47 and 50 (*p* = 0.01, 0.02, 0.02, 0.002 and 0.0001 respectively) consistent with phylogenetic observations above. The recombination event 21 was highly enriched in the type 2 genomes (*p* = 8.97*e* − 10). We then compared the number of recombinant and non-recombinant genomes between EBV types (Table 2). We further classified the EBV genomes as recombinant based on the presence of 1 or more recombinant segments and as non-recombinant genomes based on the absence of recombinant segments within the genomes. The viral type was significantly associated with the recombination status of the genomes (*p* = 0.011) with more recombinant genomes reported among the type 1 genomes (71.8%). We then compared the number of recombinant portions per genome between EBV types (Fig. 2A). Type 1 viruses had an average of 2.16 events per genome while type 2 viruses had 1.03 events per genome. Consequently, type 1 genomes reported significantly more recombination events (*p* = 6.4*e* − 06). The majority of these events (78.6%) were present in one EBV type with just 7.1% (2/28) of the events found in genomes from both viral types. Additionally, the overall number of different events in type 1 viruses was significantly higher than the type 2 viruses (*p* = 1*e* − 05) (Additional file 3: Fig. S3).

**Table 1:** EBV type association with unique recombination events

Recombination event	CDS cut by start breakpoint	CDS cut by end breakpoint	Frequency in type 1 (%)	Frequency in type 2 (%)	*p value*
21	BORF1, BORF2	BRLF1	0/56 (0)	20/30 (63)	**8.97e − 10**
28	BRLF1	BKRF2	11/56 (20)	0/30 (0)	**0.01**
37	EBNA3B	BGLF1, BGLF4	9/56 (16)	0/30 (0)	**0.02**
38	BNRF1	BOLF1, BPLF1	9/56 (16)	0/30 (0)	**0.02**
47	BRLF1, BZLF1	BDLF3.5, BDLF4	22/56 (39)	0/30 (0)	**0.002**
50	LMP2A, LMP2B	EBNA2	20/56 (36)	0/30 (0)	**0.0001**

*eBL* endemic Burkitt lymphoma, *CDS* coding sequence

Bold text indicates a statistically significant difference with a *p value* < 0.05. All groups’ proportions were compared using Fisher’s exact test

**Table 2:** Factors associated with recombination

Characteristic	Total	Recombinant genomes (%)	Non-recombinant Genomes (%)	*p value*
N	86	71 (82.6)	15 (17.5)	
*Viral type*				
Type 1	56	51 (71.8)	5 (33.3)	**0.011**^**a**^
Type 2	30	20 (28.2)	10 (66.6)	
*BL status*				
eBL	54	48 (67.6)	6 (40)	0.086^a^
Healthy	32	23 (32.4)	9 (60)	

*eBL* endemic Burkitt lymphoma

Bold text indicates a statistically significant difference with a *p value* < 0.05. Groups’ proportions were compared using ^a^Pearson’s Chi-square

### The locations of recombination breakpoints along EBV genome

**Fig. 3:**
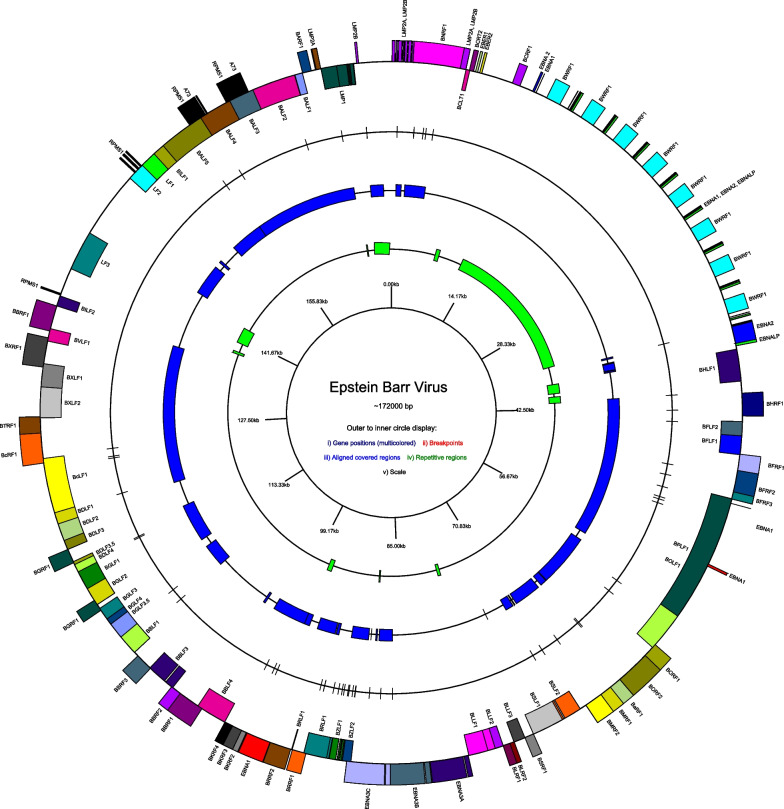
EBV genome map with positions of recombination breakpoints. From outer to inner, circles display genomic positions for (i) gene positions, (ii) breakpoints, (iii) aligned covered regions, (iv) repetitive regions, and (v) scale. Genes are color-coded based on the gene exons. Genes on the outside are transcribed clockwise and the inner are counterclockwise. This figure was drawn by GenomeVx

**Fig. 4:**
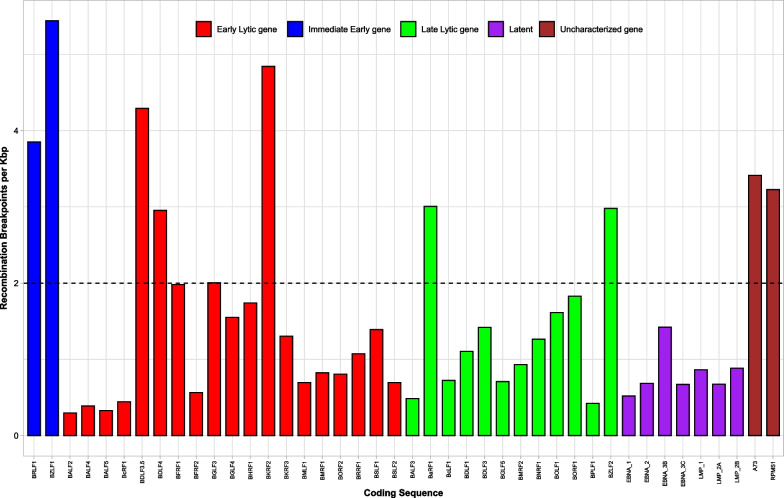
Distribution of recombination breakpoints in EBV coding sequences. Abbreviations. Kbp, Kilobase pair; CDS, Coding Sequence. Each colored bar represents an EBV gene. The total number of CDS = 42. Of the 42 CDS, 6 (14.3%) are latent genes and 36 (85.7%) are lytic genes. The bars are colored according to the classification of the genes: EBV lytic cycle,Blue; Immediate Early genes, Red; Early lytic genes, Green; Late Lytic genes, Purple; Latent genes, Brown; and Uncharacterized genes, Maroon. The black dotted strip denotes the 3rd interquartile for the number of recombination breakpoints per Kbp for all the 42 genes (2.0)

EBV has been shown to exhibit a heterogeneous pattern of recombination along its genome [33] and therefore we sought to find out where along the genome these recombination events occur (Fig. 3). The identified event breakpoints appeared to cluster at specific genomic locations. One cluster of breakpoints that stood out was located within the *BZLF1* and *BRLF1* exons. These recombination breakpoints were found in 42 protein-coding genes (Fig. 4). Of the 42 genes, only 7 (16.67%) were genes of the latent EBV cycle. Investigating further, 19 were early lytic genes, 12 were late lytic genes and interestingly, the 2 immediate early genes i.e. *BZLF1* and *BRLF1*. In exploring the recombination breakpoints per kilobase pair (Kbps) for protein-coding genes, we made comparisons among the 42 EBV genes with varying lengths. The mean number of recombination breakpoints per Kbp for all the genes was 1.58 (range 0.30–5.44). A total of 9 genes; *BZLF1*, *BRLF1*, *BDLF3.5*, *BDLF4, BaRF1, BKRF2, BZLF2, A73,* and *RPMS1* had their count of recombination breakpoints per Kbp above the 3rd interquartile (2.0). Of these 9 genes with elevated numbers of recombination breakpoints per Kbp, 7 (77.8%) were known lytic genes while 2 (22.2%) were uncharacterized genes.

### Recombination signatures associated with eBL

Recombination events have the capacity to dramatically reassociate variation and create variant profiles that may en masse affect virulence or the risk of eBL. One hypothesis is that recombinant genomes, in general, may have increased oncogenic potential for eBL. To investigate this, we compared the proportions of recombinant genomes and non-recombinant genomes between the healthy and the eBLs. More genomes with recombinant pieces were found among the eBLs (67.6%) but the difference was not statistically significant (*p* = 0.086) (Table 2). We thereafter compared the number of recombination events per genome between eBLs and healthy children (*p* = 0.037) (Fig. 2B). Since we have already shown differences between type 1 and type 2 viruses and association with eBL, we assessed recombinant levels between viral types separately and found no significant differences within type 1 or type 2 viruses relative to disease (type 1 genomes; *p* = 0.17 & type 2 genomes; *p* = 0.58) (Fig. 2C). However, the mean and interquartile values were greater in the eBLs (*mean* = 2.282, *range* = 2.00–3.00) compared to the healthy (*mean* = 1.882, *range* = 1.00–2.00), particularly among the type1 viruses.

It may also be possible that specific recombinant events are associated with eBL risk so we probed eBL association with recombination events (Table 3). Two recombination events were significantly enriched in the eBLs; event 47 (*OR* = 4.07, *p* = 0.038) and 50 (*OR* = 14.24, *p* = 0.012*)*. The coordinates of the breakpoints associated with these events may have biological significance that can inform their association with disease. Event 47 breakpoints are located in *BRLF1*, *BZLF1*, *BDLF3.5*, and *BDLF4* while event 50 associated breakpoints occurred within; *LMP2A*, *LMP2B, EBNA2.* Controlling EBV viral type, only event 50 was significantly enriched in the eBLs (*OR* = 12.36*, p* = 0.020) while event 47 still showed a suggestive link with eBL (*OR* = 3.31*, p* = 0.089*).*

**Table 3:** eBL association with unique recombination events

Event	CDS cut by start breakpoint	CDS cut by end breakpoint	Frequency in eBLs (%)	Frequency in healthy (%)	Without controlling for viral type		Controlling for viral type	
					OR^a^ (95% CI)	*p value*	OR^b^ (95% CI)	*p value*
47	BRLF1, BZLF1	BDLF3.5, BDLF4	16/54 (29.6)	3/32 (9.4)	4.07 (1.21–1.87)	**0.038**^**a**^	3.31 (0.99–1.58)	0.089^b^
50	LMP2A, LMP2B	EBNA2	17/54 (31.5)	1/32 (3.1)	14.24 (2.69–2.84)	**0.012**^**a**^	12.36 (2.18–2.34)	**0.020**^**b**^

*CDS* coding sequence, *eBL* endemic Burkitt lymphoma, *OR* odds ratio, *Ref* reference

Bold text indicates a statistical significance with a *p value* < 0.05. ^a^Univariate and ^b^Multivariate logistic regression was used to compute the Odds Ratios and *p values* non-significant *p value* by univariate analysis

## Discussion

In this study, we used samples from a defined population in a malaria-endemic region in Western Kenya to characterize recombination in EBV as a source of genetic diversity and for association with eBL. The majority of EBV genomes sequenced harbored one or more recombinant segments, with type 1 virus demonstrating more recombinant segments compared to type 2 genomes. Further, we show that recombinant segments shared by multiple isolates were driving a significant portion of EBV relatedness and phylogeny. Along the EBV genome, the recombination breakpoints were non-uniform and were enriched at specific genome sites, especially within lytic genes. Importantly, some of these type-specific recombinant segments were enriched among viral isolates from eBL patients. Viral recombination has been long-recognized and the molecular mechanism is thought to require two or more EBV genomes to co-infect a host cell and exchange genomic segments [31] thus multiple EBV infections and reinfections within our population may fuel the exchange of genomic segments. The extent of recombnatoin suggests that the human host immune response insufficiently defends against subsequent EBV infections. Such repeated infections may be prone in Western Kenya where children contract EBV at an early age [55] and experience repeated exposure to *Pf* infection [9] known to activate the polyclonal expansion of the B cells, causing EBV reactivation and a spike in peripheral blood viral loads. These factors may help drive recombination [56] and expand EBV’s population diversity, which could confound host immune surveillance.

Further, we demonstrated that recombinant segments shared by multiple isolates were a major driver of the pattern of nucleotide variation and thus relatedness within EBV phylogeny. Thus, successive recombination events occur frequently enough to drive these patterns without being so frequent as to lead to homogenization. Common phylogenetic classifications of EBV are characterized by clustering of isolates [11, 22] and interestingly, our study showed new evidence that recombinant segments may be a major driver of such relatedness.

Importantly, our study provides the first comparison of recombination between EBV type 1 and type 2. We found that type 1 genomes have accumulated and preserved more recombination events which may be the consequence of different recombination rates and/or larger viral population sizes [21, 57]. Bearing more recombinant segments, our findings are consistent with previous observations that EBV type 1 bears greater nucleotide diversity compared to EBV type 2 [4, 21]. Additionally, our observations speculate on the possible contribution of recombination events on the differential mutational loads and tumorigenicity of EBV types.

The impact of recombination on genes showed enrichment of recombination breakpoints in EBV lytic genes. This observation may be explained by the molecular mechanism of recombination which is thought to be intimately linked to the EBV lytic phase, characterized by episodes of lytic reactivation and replication. Our comparison of recombination rates across EBV genomic sites was however limited to about 51% of the whole EBV genome, which had reliable nucleotide content. While this may cause the risk of missing some recombination sites in genomic locations not analyzed, it allowed the study to reliably call recombination events and avoid inferring artificial nucleotide diversity.

Since recombination appears to drive patterns of EBV variation, recombination may be a source of risk variants that may alter the viral phenotype and virulence to augment the risk of eBL pathogenesis. Our comparison of recombination patterns between the viral isolates from the healthy and eBL counterparts reveals type-specific recombination patterns that were enriched among the eBLs. Recombination breakpoints were enriched in coding regions of biologically important EBV genes such as the *BZLF1* and *BRLF1*, a phenomenon that could change the antigenic determinants of such viral proteins and facilitate immune escape from the human host as was previously reported in herpesviruses [32, 50–52]. In this study, however, the possible associations between EBV recombinant proportions, their breakpoints, and eBL were largely explained by their enrichment among the EBV type 1 isolates. While EBV type 1 has been shown to be associated with eBL in a previous study [21] the exact mechanism(s) is still being investigated. Our study provided insights into novel EBV variation profiles that may contribute to eBL pathogenesis.

In summary, our analyses of recombination in EBV genomes from our well-defined population of healthy and eBL individuals from Western Kenya suggest that recombination is a frequent occurrence and major driver of variation in the EBV population with potential associations with oncogenesis. Further comparative and in vitro studies involving EBV complete genomes with representative sampling globally are needed to understand the complete and global role of recombination in EBV and disease.

## Supplementary Information

**Additional file 1.** Recombination Events in Plasma-Tumor Replicates A.) Abbreviation: eBL, endemic Burkitt lymphoma. Phylogenetic Tree of 6 plasma and tumor replicates. Each plasma and tumor replicate has a unique colour e.g. eBL-Tumour-0036 and eBL-Plasma-0036 are colored in red. B.) The figure illustrates a comparison of recombination patterns of 4 plasma-tumor replicates (35, 37, 38, and 39). Each side-by-side bar represents a unique event in a plasma and tumor isolate.

**Additional file 2.** Frequency of Distinct Recombination Events: Each colored bar represents a distinct genomic recombination event as reported by RDP4. Each number on the x-axis is the name of each distinct genomic recombination event as coded by RDP4. The number of recombination events retained after filtering well-supported recombination events = 28.

**Additional file 3.** Number of Distinct Recombination Events Stratified by Viral Type: Center Lines represent medians, with lower and upper boundaries of the boxes representing first and third quartiles respectively. A wilcoxon test was performed and P-value.

**Additional file 4.** Dataset of 86 Genomes: Comprised of 54 confirmed eBL cases and 32 geographically matched healthy children plus their corresponding data which includes the age, viral type, and gender of the participants.

**Additional file 5.** RDP4 Output: 28 Recombination events detected by all six RDP4 methods. Bears details of recombination events detected coded with unique numbers, sequences with evidence of such events, and the coordinates of the corresponding breakpoints in the MSA.

**Additional file 6.** Demographic Characteristics of Study Participants. Abbreviation: eBL, endemic Burkitt lymphoma. Bold text indicates a statistically significant difference with a P-value.

**Additional file 7.** Trimmed Multiple Sequence Alignment (MSA): Represents the output of the MSA of 86 genomes with MAFFT followed by MSA trimming with Gblocks. The MSA covers ~51% (88 kbp) of the 172kbp EBV genome.

## Data Availability

The dataset analyzed in this study is available at the European Nucleotide Archive (http://www.ebi.ac.uk/ena), under the study accession no. ERP122181. All data generated during the analysis are included in this published article [and its supplementary information files].

## Abbreviations

AID: Activation induced deaminase
BART: BamHI rightward transcript
BHRF1: Bam HI-H rightward fragment 1
C terminal: Carboxyl terminal
CDS: Coding sequence
DNA: Deoxyribonucleic acid
EBER: Epstein–Barr virus encoded RNA
eBL: Endemic Burkitt lymphoma
EBNA: Epstein–Barr nucleotide antigen
EBNA-LP: Epstein–Barr nuclear antigen-L protein
EBV: Epstein–Barr virus
ENA: European nucleotide archive
GC: Germinal Centre
HCMV: Human cytomegalovirus
HHV4: Human herpesvirus 4
HSV1: Herpes simplex virus 1
IGV: Integrative genome viewer
JOORTH: Jaramogi Oginga Odinga Teaching and Referral Hospital
Kbp: Kilobase pairs
KEMRI: Kenya Medical Research Institute
LCV: Lymphocryptovirus
LMP: Latent membrane protein
MAFFT: Multiple alignment by fast Fourier transform
MCMV: Murine cytomegalovirus
MEGA: Molecular evolutionary genetics analysis
MSA: Multiple sequence alignment
NJ: Neighbour joining
NPC: Nasopharyngeal carcinoma
OR: Odds ratio
ORFs: Open reading frames
Pf: *Plasmodium falciparum*
RPD4: Recombination detection program 4
SNP: Single nucleotide polymorphism
SSA: Sub-Saharan Africa
UMMS: University of Massachusetts Medical School
USA: United States of America
