# Supplementary material for: Profiling genome-wide recombination in Epstein Barr virus reveals type-specific patterns and associations with endemic-Burkitt lymphoma
Source: Virol J. 2022 Dec 8;19:208. doi: 10.1186/s12985-022-01942-8 (PMC9733152; doi:10.1186/s12985-022-01942-8)
Supplement: Supplementary file 1 — Additional file 1. Recombination Events in Plasma-Tumor Replicates A.) Abbreviation: eBL, endemic Burkitt lymphoma. Phylogenetic Tree of 6 plasma and tumor replicates. Each plasma and tumor replicate has a unique colour e.g. eBL-Tumour-0036 and eBL-Plasma-0036 are colored in red. B.) The figure illustrates a comparison of recombination patterns of 4 plasma-tumor replicates (35, 37, 38, and 39). Each side-by-side bar represents a unique event in a plasma and tumor isolate. [file 12985_2022_1942_MOESM1_ESM.pdf]

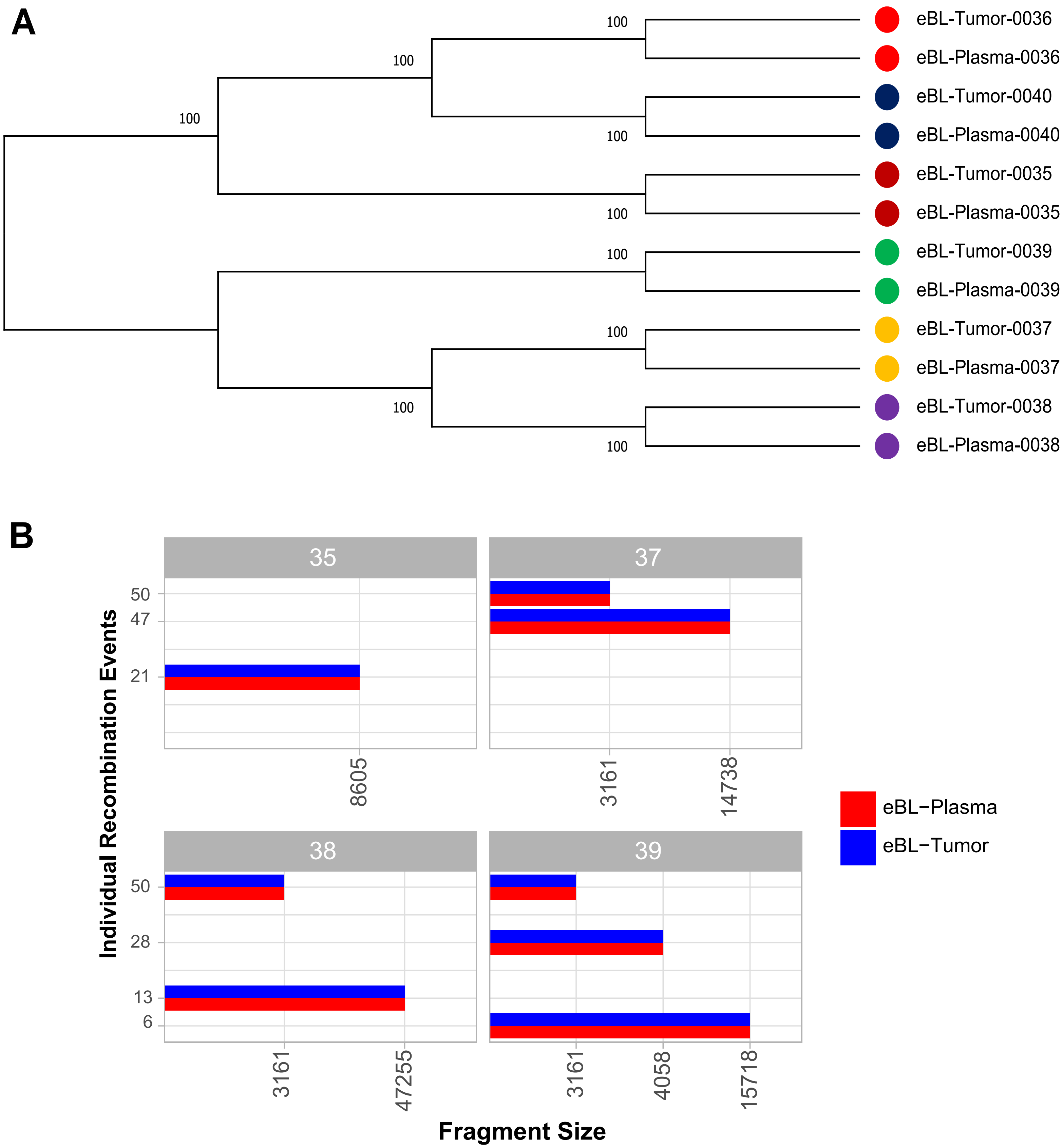

Supplementary material, Figure 1. Recombination Events in Plasma-Tumor Replicates **A.)** Abbreviation: eBL, endemic Burkitt lymphoma. Phylogenetic Tree of 6 plasma and tumor replicates. Each plasma and tumor replicate has a unique colour e.g. eBL-Tumour-0036 and eBL-Plasma-0036 are colored in red. **B.)** The figure illustrates a comparison of recombination patterns of 4 plasma-tumor replicates (35, 37, 38, and 39). Each side-by-side bar represents a unique event in a plasma and tumor isolate.
