# Supplementary material for: Profiling genome-wide recombination in Epstein Barr virus reveals type-specific patterns and associations with endemic-Burkitt lymphoma
Source: Virol J. 2022 Dec 8;19:208. doi: 10.1186/s12985-022-01942-8 (PMC9733152; doi:10.1186/s12985-022-01942-8)
Supplement: Supplementary file 2 — Additional file 2. Frequency of Distinct Recombination Events: Each colored bar represents a distinct genomic recombination event as reported by RDP4. Each number on the x-axis is the name of each distinct genomic recombination event as coded by RDP4. The number of recombination events retained after filtering well-supported recombination events = 28. [file 12985_2022_1942_MOESM2_ESM.pdf]

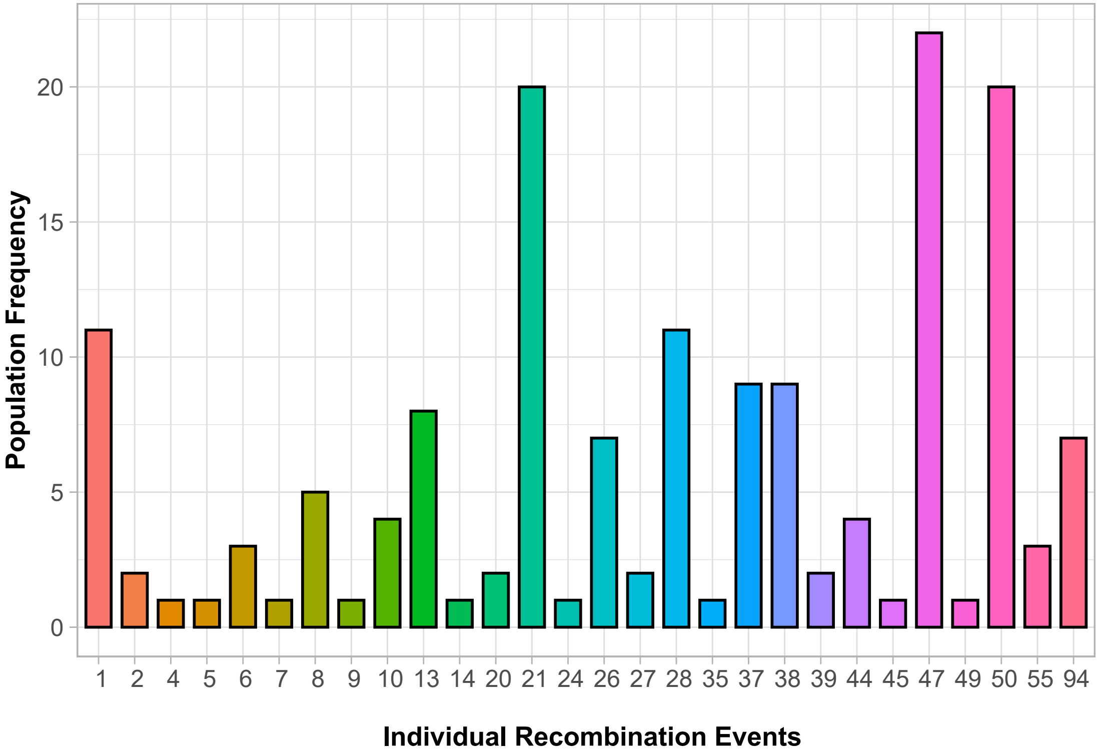

Supplementary material, Figure 2. Frequency of Distinct Recombination Events: Each colored bar represents a distinct genomic recombination event as reported by RDP4. Each number on the x-axis is the name of each distinct genomic recombination event as coded by RDP4. The number of recombination events retained after filtering well-supported recombination events=28.
