# Supplementary material for: Profiling genome-wide recombination in Epstein Barr virus reveals type-specific patterns and associations with endemic-Burkitt lymphoma
Source: Virol J. 2022 Dec 8;19:208. doi: 10.1186/s12985-022-01942-8 (PMC9733152; doi:10.1186/s12985-022-01942-8)
Supplement: Supplementary file 3 — Additional file 3. Number of Distinct Recombination Events Stratified by Viral Type: Center Lines represent medians, with lower and upper boundaries of the boxes representing first and third quartiles respectively. A wilcoxon test was performed and P-value. [file 12985_2022_1942_MOESM3_ESM.pdf]

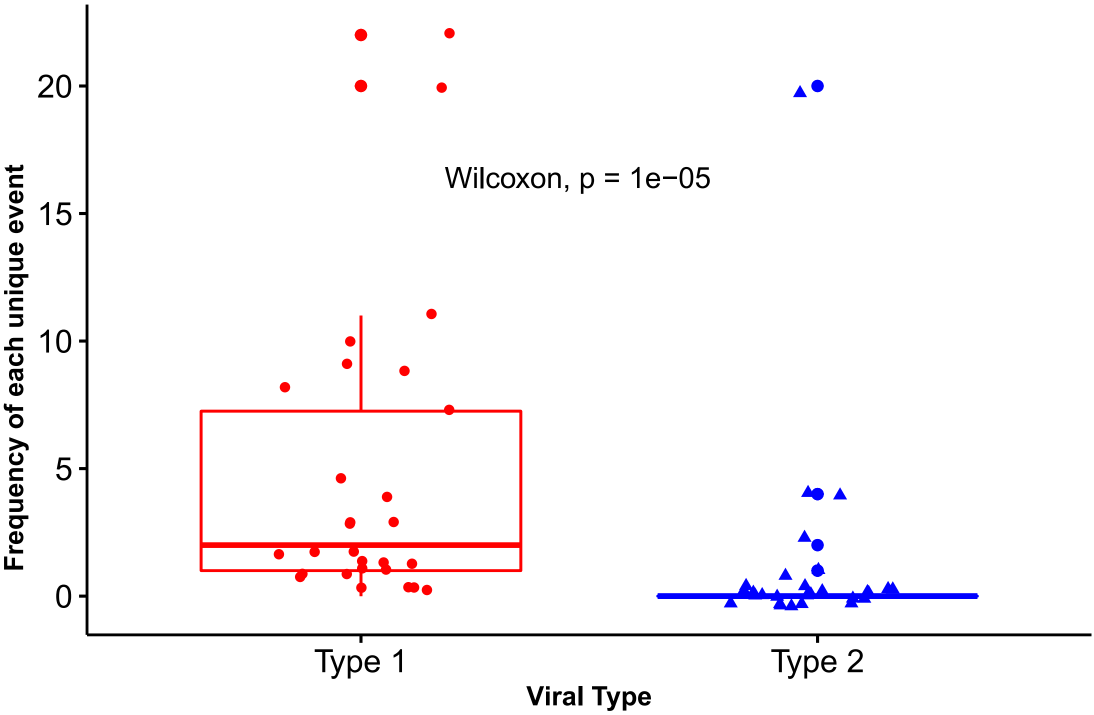

Supplementary material, Figure 3. Number of Distinct Recombination Events Stratified by Viral Type: Center Lines represent medians, with lower and upper boundaries of the boxes representing first and third quartiles respectively. A wilcoxon test was performed and P-value < 0.05 was considered significant.
