# Supplementary material for: Profiling genome-wide recombination in Epstein Barr virus reveals type-specific patterns and associations with endemic-Burkitt lymphoma
Source: Virol J. 2022 Dec 8;19:208. doi: 10.1186/s12985-022-01942-8 (PMC9733152; doi:10.1186/s12985-022-01942-8)
Supplement: Supplementary file 6 — Additional file 6. Demographic Characteristics of Study Participants. Abbreviation: eBL, endemic Burkitt lymphoma. Bold text indicates a statistically significant difference with a P-value. [file 12985_2022_1942_MOESM6_ESM.docx]

**Supplementary material, Table 3. Demographic Characteristics of Study Participants**

| Characteristic | | Total | eBL (%) | Healthy (%) | *P-Value* |
| --- | --- | --- | --- | --- | --- |
| Participants | | 86 | 54 (62.8) | 32 (37.2) |  |
| Gender | Female | 28 | 14 (35.9) | 14 (43.8) | **0.1424^a^** |
|  | Male | 58 | 40 (74.1) | 18 (58.2) |  |
| Age Group | 0-4 | 39 | 10 (18.5) | 29 (90.6) | **5.735e-11^b^** |
|  | 5-9 | 34 | 31 (57.4) | 3 (9.4) |  |
|  | 10-14 | 13 | 13 (24.1) | 0 (0) |  |
| Viral Type | Type 1 | 56 | 39 (72.2) | 17 (53.1) | **0.1183^a^** |
|  | Type 2 | 30 | 15 (27.8) | 15 (46.9) |  |

Abbreviation: eBL, endemic Burkitt lymphoma. Bold text indicates a statistically significant difference with a *P-value*<0.05. Groups’ proportions were compared using ^a^Pearson’s Chi-square and ^b^Fisher exact tests.

**Supplementary material, Trimmed Multiple Sequence Alignment:** This represents the output of the MSA of 86 genomes with MAFFT followed by MSA trimming with Gblocks. The MSA covers ~51% (88 kbp) of the 172kbp EBV genome.
